# A model for rapid, active surveillance for medically-attended acute gastroenteritis within an integrated health care delivery system

**DOI:** 10.1371/journal.pone.0201805

**Published:** 2018-08-03

**Authors:** Mark A. Schmidt, Holly C. Groom, Allison L. Naleway, Christianne Biggs, S. Bianca Salas, Kayoko Shioda, Zachary Marsh, Judy L. Donald, Aron J. Hall

**Affiliations:** 1 Center for Health Research, Kaiser Permanente Northwest, Portland, Oregon, United States of America; 2 Oregon State Public Health Laboratory, Public Health Division, Oregon Health Authority, Hillsboro, Oregon, United States of America; 3 Division of Viral Diseases, Centers for Disease Control and Prevention, Atlanta, Georgia, United States of America; Universita degli Studi di Parma, ITALY

## Abstract

**Background:**

This study presents a novel methodology for estimating all-age, population-based incidence rates of norovirus and other pathogens that contribute to acute gastroenteritis in the United States using an integrated healthcare delivery system as a surveillance platform.

**Methods:**

All cases of medically attended acute gastroenteritis within the delivery system were identified from April 1, 2014 through September 30, 2016. A sample of these eligible patients were selected to participate in two phone-based surveys and to self-collect a stool sample for laboratory testing. To ascertain household transmission patterns, information on household members with acute gastroenteritis was gathered from participants, and symptomatic household members were contacted to participate in a survey and provide stool sample as well.

**Results:**

54% of individuals who met enrollment criteria agreed to participate, and 76% of those individuals returned a stool sample. Among household members, 85% of eligible individuals agreed to participate, and 68% of those returned a stool sample. Participant demographics were similar to those of the eligible population, although minority racial/ethnic groups were somewhat underrepresented in the final sample.

**Conclusions:**

This study demonstrates the feasibility of conducting acute infectious disease research within an integrated health care delivery system. The surveillance, sampling, recruitment, and data collection methods described here are broadly applicable to conduct baseline and epidemiological assessments, as well as for other research requiring representative samples of stool specimens.

## Introduction

In the United States, Acute Gastroenteritis (AGE) accounts for more than 179 million cases of illness and 600,000 hospitalizations on an annual basis [1]. AGE, which is caused by a variety of both infectious and noninfectious agents, affects all age groups; however, it disproportionally affects infants and young children. Virtually all children experience AGE by five years of age, with highest rates of infection occurring in those 6–24 months of age [2]. Since the introduction of the rotavirus vaccine program in 2006 [3–5], norovirus has emerged as the leading cause of AGE for all age-groups, estimated to cause 19–21 million illnesses and 570–800 deaths in the United States each year [6]. Those 65 years and older are at greatest risk for norovirus-associated deaths, while children younger than 5 years old experience the highest rates of norovirus-associated medical encounters: an estimated 1 million healthcare visits annually [6, 7].

Precise estimates of all-age, population-based incidence rates of norovirus and other viral pathogens that contribute to AGE in the U.S. are needed to allow for an accurate assessment of the impact of a future vaccine [2, 8, 9]. However, incidence of AGE, particularly for those illnesses caused by laboratory-confirmed viral pathogens such as norovirus, has been difficult to estimate for several reasons. First, neither AGE in general, nor its associated viral pathogens, are reportable to health authorities. Second, a historic lack of widely available diagnostic assays has led to scant laboratory data to generate these estimates [10]. Consequently, previous estimates of population-based incidence of AGE and its associated viral pathogens within the U.S. have largely been derived from targeted surveillance among the pediatric population, testing of available clinical stool specimens collected for bacterial testing, and extrapolations of attributable proportion data from other industrialized countries [1, 7, 11, 12]. These methods did not rely on population-based, representative samples and thus may not have resulted in accurate estimates of the true incidence of disease caused by AGE pathogens. Further, the role of household transmission in AGE incidence is poorly understood, complicating efforts to identify and address risk factors associated with increased transmission in the household setting.

To address these needs, we recently established the first all-age, population-based assessment of medically-attended AGE (MAAGE) in the United States through the collection of survey data and stool samples of patients and household members (HHM) using an integrated health care delivery system, an organized healthcare system that coordinates care, offers a continuum of primary and specialty health services, and uses a comprehensive electronic health record to document medical information for its enrolled population, as a surveillance platform. This methodology is applicable to research on vaccine effectiveness for AGE viruses and other conditions, as well as for population-based research on other gastrointestinal health issues. In this paper, we describe the surveillance procedures and methods implemented to conduct this assessment as a model for conducting active, population-based surveillance.

## Methods

### Methods overview

We implemented our surveillance program within Kaiser Permanente Northwest (KPNW), an integrated health care delivery system with a well-defined population of approximately 605,000 members, as of June 2018. We followed all enrolled members of KPNW from April 1, 2014 through September 30, 2016 to identify MAAGE-related encounters, and sought to recruit an age-stratified, representative sample of those to submit a stool sample and complete baseline and follow-up surveys. Additionally, we obtained information about all HHM of participants with a MAAGE encounter. HHMs who had experienced AGE symptoms within 7 days of their family member’s enrollment or follow-up questionnaire were recruited, regardless of KPNW membership status, to submit a stool specimen and complete a survey. Data from the KPNW electronic health record (EHR) was used to supplement questionnaire and stool sample information in analyses.

### Surveillance site

KPNW membership comprises roughly 24% of, and is demographically similar to, the total population of the underlying catchment area of Northwest Oregon and Southwest Washington (Table 1). KPNW maintains a comprehensive EHR system that includes all aspects of members’ health care, including health plan enrollment, encounters, diagnoses, procedures, prescription orders and fills, and laboratory testing and results. We collaborated with the Oregon State Public Health Laboratory (OSPHL) to conduct additional laboratory testing for viral agents of AGE in stool specimens submitted by study participants.

**Table 1:** Comparison of demographic and insurance status characteristics.

Characteristic	Portland ACS 2015^a^	KPNW 2016
**Gender (%)**		
Male	49	48
Female	51	52
**Age (yrs.) (%)**		
<20	21	19
20–44	43	36
45–64	25	28
≥65	11	17
**Race (%)**		
White	78	75
Black	6	2
American Indian	1	1
Asian	8	4
Hawaiian/Pacific Islander	1	1
Other	3	17
**Ethnicity (%)**		
Hispanic/Latino	10	4
**Insurance type (%)**		
Medicare	13	19
Medicaid	19	7
Neither	79	74

^a^ACS: American Community Survey

### Case identification and sampling

We developed a daily, automated extraction and sampling system to identify all MAAGE encounters that had occurred during the previous 24-hour period. Per our case definition, we defined a MAAGE encounter as any email visit, telephone visit, video visit (beginning in 2016), ambulatory care clinic visit, urgent care visit, emergency department visit, or hospital admission within the KPNW health care delivery system at which a diagnosis of AGE was recorded within the EHR, either through *Chief Complaint* (for telephone encounters) or *International Classification of Disease*, *9*^*th*^ (ICD-9, used from 4/1/2014-9/30/2015) or *10*^*th*^ *Revision* (ICD-10, used from 10/1/2015-9/30/16) codes (for all other encounters) (Table 2) [13]. We defined a discrete MAAGE episode as beginning at the time of an identified MAAGE-related encounter and including all subsequent MAAGE-related encounters occurring within 30 days of one another. In other words, the MAAGE episode ended when at least 30 days passed after the last MAAGE-related encounter.

**Table 2:** ICD-9/10 codes used to define MAAGE encounters.

Diagnosis	ICD-9	ICD-10
**Cause unspecified**		
Presumed infectious	009.0–009.3	A09
Presumed noninfectious	558.9	K52.9
Symptom: Diarrhea NOS	787.91	
**Cause specified**		
**Viral**	008.61–008.8	A08.0-A08.5
Rotavirus	008.61	A08.0
Adenovirus	008.62	A08.2
Norwalk	008.63	A08.1
Other viral enteritis	008.64–008.69	A08.3, A08.5
Other not elsewhere classified	008.8	A08.4
**Bacterial**	001.0–005.9, 008.0–008.5	A00.0-A05.9
Cholera	001.0–00.9	A00.0-A00.9
Typhoid/Paratyphoid	002.0–002.9	A01.0-A01.4
Salmonella	003.0–003.9	A02.0-A02.9
Shigella	004.0–004.9	A03.0-A03.9
Other bacterial food poisoning	005.0–005.9	A05.0-A05.9
E. coli	008.0	A04.0-A04.4
Other/unspecified bacteria	008.1–008.5	A04.5-A04.9
**Parasitic**	006.0–006.2, 006.9–007.9	A06.0-A07.9
Ameba	006.0–006.2, 006.9	A06.0-A06.2
Other protozoal	007.0–007.9	A07.0-A07.9

Once encounters were abstracted, we applied a sampling scheme to identify individuals who were potentially eligible for recruitment. Because MAAGE disproportionally affects the youngest and eldest age groups, we opted to oversample from those younger than 5 years and those 75 years and older to maximize the precision of the incidence estimates among these critical populations. Consequently, we sampled 100% of potentially eligible individuals in these two age groups and 35% of those from the remainder (5–17 years, 18–44 years, 45–64 years, and 65–74 years). We excluded individuals who were previously eligible for recruitment within a given surveillance year and those with documented diagnostic codes for conditions associated with chronic diarrhea.

### Recruitment and enrollment

**Fig 1:**
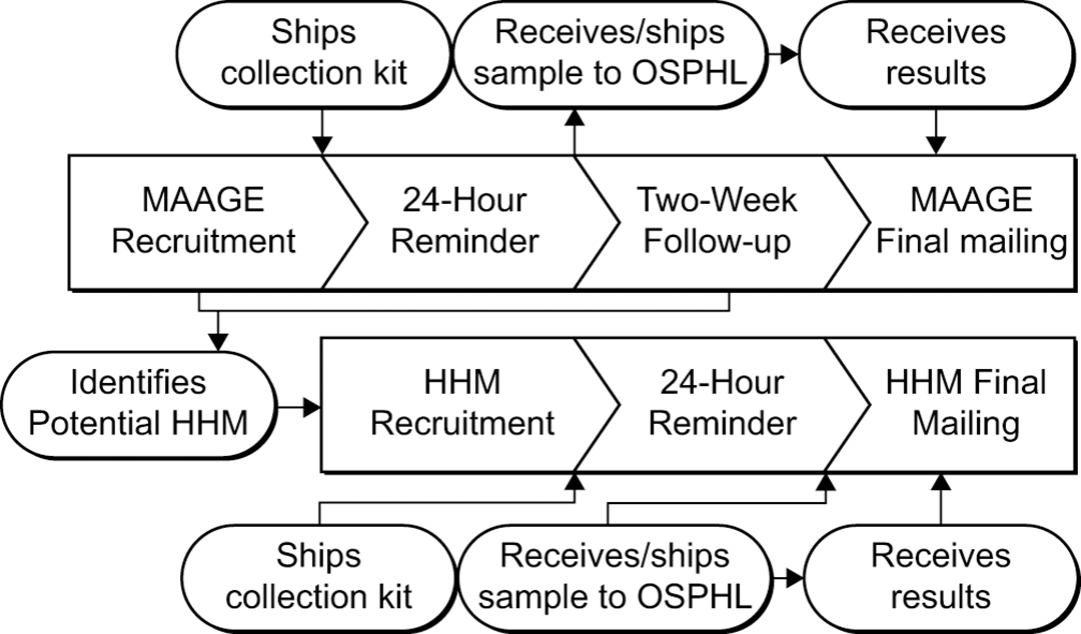
Overview of MAAGE surveillance design and process. ^a^MAAGE: Medically-attended acute gastroenteritis. ^b^OSPHL: Oregon State public health lab. ^c^HHM: Household member.

An overview of the recruitment and enrollment process is shown in Fig 1. All recruitment was conducted by dedicated staff within the KPNW Center for Health Research (CHR). Six days per week, Monday through Saturday, recruitment staff would attempt to contact recruitment-eligible participants or their parent or legal guardian (for those younger than 18 years of age) by telephone. One telephone attempt was made to each potentially eligible participant per day. Potential participants were automatically removed from recruitment lists and classified as *inactive* after three days of unsuccessful contact attempts by recruiters. We limited the recruitment window in order to maximize our ability to obtain stool specimens from participants within ten days of their MAAGE encounter and thus minimize false negative results, This approach is consistent with methodologic approaches of previous studies [7, 14]. For those successfully contacted, recruiters conducted informed consent procedures and administered a brief screening survey to ascertain enrollment eligibility to consenting individuals.

### Case definition

Eligibility was contingent on having a health encounter with an AGE-associated ICD9, ICD10, or chief complaint code (Table 2) and having symptoms that include vomiting (≥1 episode within 24 hours), and/or diarrhea (≥3 episodes within 24 hours). Individuals with diarrhea due to a medical condition that causes chronic diarrhea (i.e., Crohn’s disease, ulcerative colitis, inflammatory bowel disease, or abdominal or colorectal cancer), as confirmed during telephone surveys, were still eligible if they have had ≥1 vomiting episode within 24 hours. We defined participants as *ineligible* if they were deceased, in hospice care, did not speak English, were cognitively impaired, or self-reported having a condition associated with chronic diarrhea without experiencing recent vomiting (i.e., participants with chronic diarrhea conditions were eligible if they reported recent vomiting). Eligible participants were then given a detailed description of the study requirements. We defined participants as *refused* if they indicated an inability or unwillingness to fully participate in the study, including completing two surveys and submitting a stool specimen for testing. For the remainder who *agreed to participate*, recruitment staff administered an initial enrollment survey and provided information about stool specimen collection and return. We classified participants who completed the enrollment survey and returned a stool specimen as *enrolled*; the remainder we considered *lost to follow-up*. While participants had to agree to complete the two-week follow-up survey in order to participate, we nonetheless considered those who had an enrollment survey completed and a stool sample received by study staff but who did not complete the follow-up survey as *enrolled*. As compensation for their time to participate in study-related tasks, we provided participants who completed both surveys and who submitted a stool specimen for testing with a $20 gift card to a local retailer.

### Household member (HHM) recruitment

We collected information about HHM of MAAGE participants during both the baseline and two-week follow-up surveys. At baseline, participants were asked to provide the age and sex of all individual members of their household, whether each household member had recently experienced AGE symptoms and, if so, the date of onset of vomiting and/or diarrhea. During the two-week follow-up survey, we asked whether any of the identified household members had subsequently developed symptoms of AGE and, if so, the date of onset. We considered HHM with onset within the seven days prior to either survey as potentially eligible for recruitment and obtained their contact information from the MAAGE participant. The same recruitment procedures used for MAAGE participants were used to recruit potentially eligible HHMs. We classified HHMs who completed the enrollment survey and returned a stool specimen as *enrolled*. We provided enrolled HHM participants with a $10 gift card.

### Baseline and follow-up questionnaires

Baseline surveys from participants with MAAGE and enrolled HHM included demographic characteristics, information about the illness episode (i.e., symptom type, severity, and duration), sources of potential exposure (i.e., recent travel, contact with ill persons, etc.), and over-the-counter treatments. Baseline surveys from participants with MAAGE included additional information about household composition, including number of household members, age and sex of each member, and whether household members were also ill. Two-week follow-up surveys from participants with MAAGE included additional information about illness episode (i.e., ongoing symptoms post-baseline survey or recurrence of symptoms) and information on whether previously-disclosed household members subsequently developed illness. HHM did not complete a two-week follow-up survey.

### Stool sample collection and return

**Fig 2:**
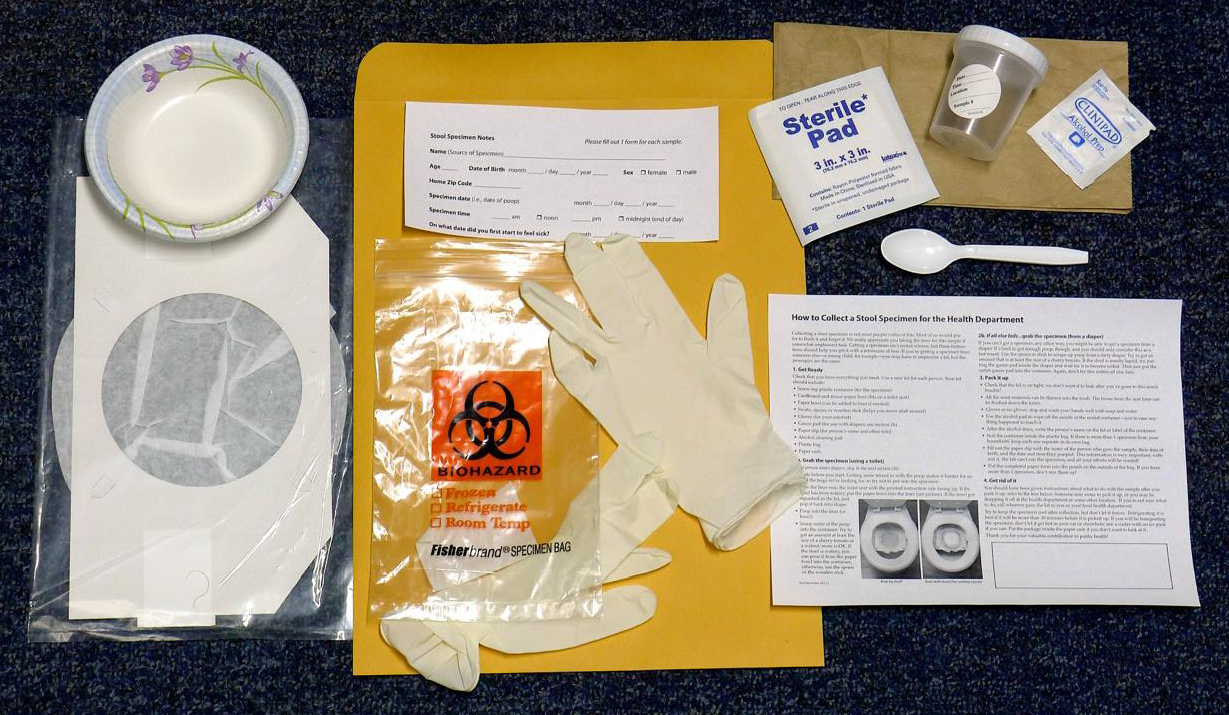
Stool sample collection kit provided to enrolled MAAGE study participants. Stool sample collection kit and information sheet (kit provided by OHPL)
• Screw-top plastic container (for the specimen). Collection container will have a label for Health Record Number (HRN), Name, date of birth (DOB), date of collection, time of collection. Recruitment staff will include member’s HRN, Name and DOB before sending kit to participant.• Card board-and-tissue-paper liner (fits on a toilet seat) with paper bowl (can be added to liner if needed)• Spoon (to scoop)• Gloves• Gauze pad (for use with diapers)• Alcohol cleaning pad• Plastic specimen bag (may say "Biohazard") with absorbent towel• Paper sack **Refrigeration Kit**
• Cold pack(s)• Chill Checker button (count of 500 for randomly selected samples)• Information sheet for keeping specimen at required temperature• Sending/Returning shipping packet and information sheet• Shipping label for return sample• Shipping box

Participants self-collected stool specimens for this project. Recruitment staff sent stool collection kits to participants via daily, overnight courier service on the day of enrollment (Fig 2). Within 48 hours of enrollment, recruitment staff contacted participants to ensure receipt of stool specimen collection kits and answer any further questions about study participation and stool collection and return. We provided the participants with three return options: participant drop off at the KPNW clinical facility of their choice, return via overnight courier service, or recruitment staff pick-up (for participants residing within 15 miles of the research center). We instructed participants to keep specimens refrigerated until return and, for participants opting for return via overnight courier service, provided gel packs that were to be frozen and included in the shipping container to keep specimens cool. In a random sample of these latter specimens, we also included temperature indicators to monitor the temperature conditions of returned specimens. Once received, we refrigerated specimens until they were processed and shipped twice per week to the OSPHL for viral pathogen testing. Participants were instructed to return specimens within 7 days of collection.

### Data abstraction from EHR

We electronically abstracted clinical information from the EHR for all individuals identified with a MAAGE encounter during the surveillance period. This information included health plan enrollment, MAAGE encounter dates and associated diagnostic codes, KPNW clinical laboratory testing ordered during MAAGE encounters and testing results, medications prescribed and filled during the six months before and seven days after MAAGE encounters, rotavirus vaccination history, and other characteristics relevant to further analyses of persons with MAAGE (such as blood type, underlying chronic co-morbidities, and pregnancy).

### Surveillance and recruitment performance metrics

To assess the representativeness of the MAAGE patient and HHM participant samples, we conducted descriptive analyses of participants, as well as of those refusing to participate and ineligible for participation. We used chi-square tests to compare the demographic characteristics of recruited MAAGE patients with potentially eligible KPNW members having at least one MAAGE encounter during the surveillance period who did not participate. We also analyzed patterns of stool specimen collection and return.

### Laboratory testing

Collected specimens were refrigerated at CHR until they were shipped to the Oregon State Public Health Lab (OSPHL) on a twice weekly basis, using an established courier service. All specimens were tested for the detection of norovirus, sapovirus, astrovirus, and rotavirus RNA by using CDC developed TaqManTM real-time RT-PCR protocols (qPCR). Briefly, MagMAXTM Viral RNA Isolation Kit (ThermoFisher Scientific) and the KingFisher automated extractor were used to extract nucleic acid from stool samples using CDC-approved extraction techniques. Reverse transcription, amplification, and detection were performed on the ABI 7500 Fast-Dx real-time PCR instrument (Applied Biosystems). All qPCR runs included: no template controls for all primer and probe sets; positive template controls for primer and probe sets; and positive extraction controls to validate the extraction procedure and reagent integrity. TaqMan probes were labeled with FAM at the 5’ end and a black hole quencher (BHQ1) at the 3’ end (Biosearch Technologies). Specimens positive for the detection of norovirus, astrovirus, sapovirus and roatvirus were subsequently genotyped using sanger sequencing methods provided by CDC. Briefly; positive nucleic acid was amplified for the region of interest using the Qiagen One-Step reverse transcription (RT)-PCR kit to acquire complementary cDNA. The PCR product was analyzed on a 2% agarose e-gel. DNA bands of appropriate fragment size were selected and purified using QIAquick PCR purification kit (Qiagen). Purified DNA fragments were cycle sequenced using the DTCS Quick Start kit (Beckman) and unincorporated fluorescent nucleotides were removed by Agencourt Clean Seq (Beckman). All sequences were run on the CEQ 8000 Beckman Coulter Sequencer and then imported into Bionumerics 6.6, analyzed, and compared to genomic references provided by CDC in the CaliciNet database.

### Human subjects

This project was reviewed and approved by the KPNW Institutional Review Board (FWA00002344).

## Results

### Characteristics of participants with MAAGE

**Fig 3:**
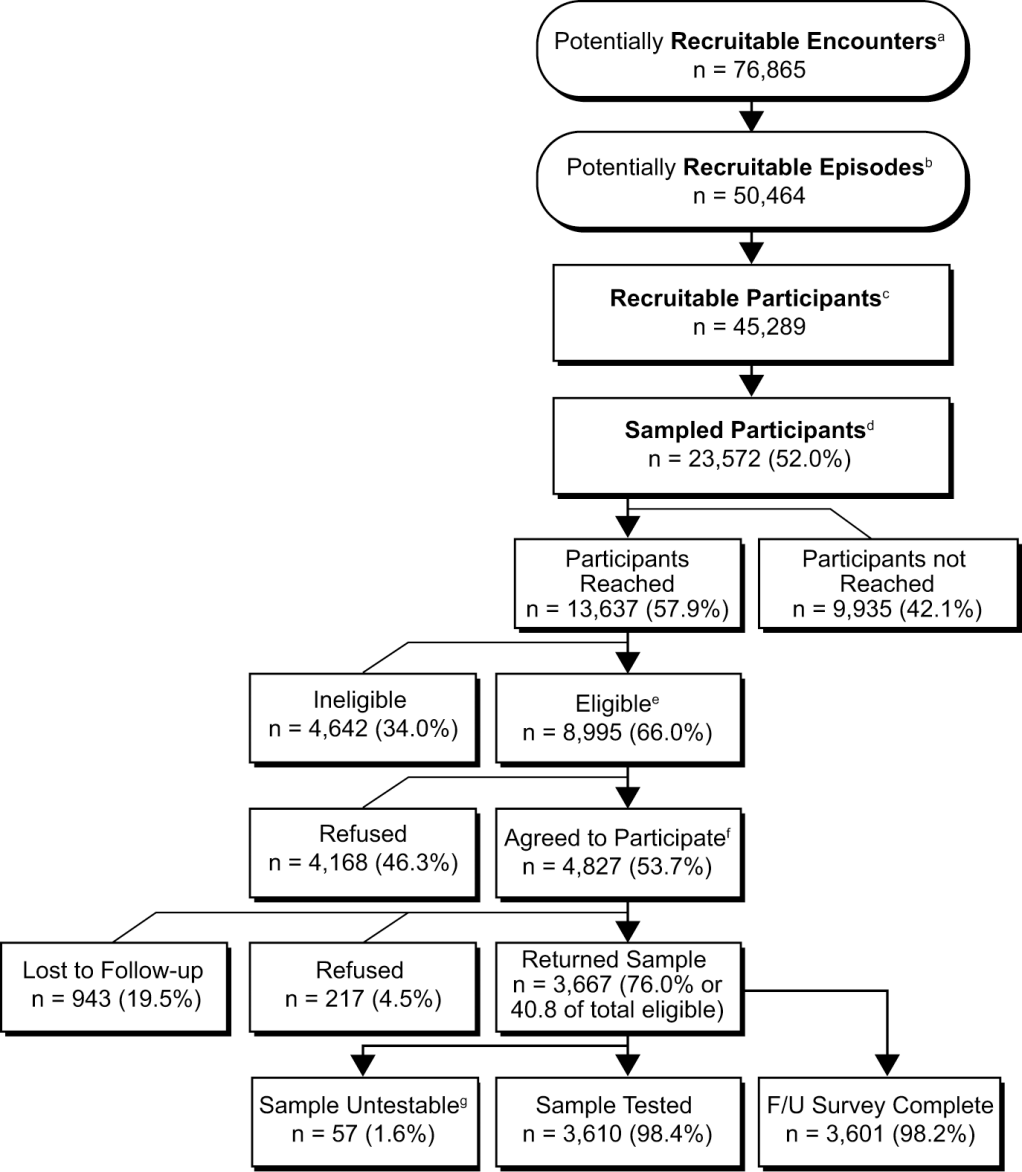
KPNW members identified with medically-attended acute gastroenteritis, April 1, 2014-September 30, 2016. ^a^Participants with a single AGE encounter per day, prior to applying exclusion criteria. ^b^Encounters occuring ≥30 days following the preceding encounter. ^c^Health plan member with ≥1 AGE encounter during the study period who meets enrollment criteria; members can have multiple events. ^d^All recruitable members aged <5 and ≥75 years of age; 35% of the following age groups: 5–17, 18–44, 45–64, 65–74. ^e^Contacted and meets all criteria for study population. ^f^Agreed to participate and completed the baseline survey. ^g^Returned samples were not pathogen-tested; 8 were rejected by the KPNW lab due to inadequate information; 49 were deemed nonviable by the Oregon State Public Health Lab.

Fig 3 provides details about the number of encounters and participants at each stage of surveillance, recruitment, enrollment, and participation. We selected 23,572 members (52% of 45,289 members eligible for recruitment) for active recruitment and successfully reached 13,637 (58%) within the required 3-day window. Of members who were not reached within this window, only 40 (<1%) were due to incorrect contact information. Of the 8,995 members who were reached and met eligibility criteria for enrollment, 4,827 (54%) agreed to participate and completed the baseline survey. Of members who agreed to participate, 100% completed the baseline survey, 76% returned a stool sample, and 75% (98% of those who returned a stool sample) completed the 2-week follow-up interview. This yielded complete data for 3,619 participants. Among 1,992 eligible MAAGE patients aged 5 years and younger, 1240 (62%) of parents agreed to participate and 922 (74%) of those parents returned a stool sample.

Of participants who returned a stool sample, the initial MAAGE encounter type was most frequently an outpatient visit (84%), with an additional 11% recruited following a remote encounter (telephone, email, or video) with their medical provider, and 5% following a hospitalized inpatient encounter.

When comparing the full eligible population (n = 45,289) to those who returned a study sample (n = 3,667), there were no significant differences in sex or geographical location (within or outside the Portland, Oregon metro area) (Table 3). However, participants were less likely to be Black (2.7% of participants vs. 3.5% of eligible members, p = 0.002), Asian (2.9% of participants vs. 3.9% of eligible members, p = 0.002), or Hispanic (7.8% of participants vs. 8.9% of eligible members, p<0.001). By design, rates of participation were significantly higher among those <5 years and >65 years.

**Table 3:** Comparison of MAAGE recruitment-eligible members and enrolled study participants, April 1, 2014-September 30, 2016.

	Recruitable members (N = 45,289^a^) n (%)	Participants (N = 3,667^b^) n (%)	p-value
**Age, in years**			**< .001**
<5	4,365 (9.6)	922 (25.1)	
5–17	4,120 (9.1)	265 (7.2)	
18–44	13,876 (30.6)	561 (15.3)	
45–64	11,815 (26.1)	723 (19.7)	
65–74	6,167 (13.6)	501 (13.7)	
≥75	4,946 (10.9)	695 (19.0)	
**Sex**			0.371
Female	27,646 (61.0)	2,211 (60.3)	
Male	17,643 (39.0)	1,456 (39.7)	
**Race**			**0.001**
White	36,184 (87.5)	3,032 (88.8)	
Black	1,449 (3.5)	93 (2.7)	
Asian	1,601 (3.9)	97 (2.8)	
One other race	789 (1.9)	66 (1.9)	
≥1 race	1,329 (3.2)	128 (3.7)	
**Ethnicity**			**< .001**
Hispanic	4,019 (8.9)	285 (7.8)	
Non-Hispanic	18,365 (40.6)	1,710 (46.6)	
Unknown	22,905 (50.8)	1,672 (45.6)	
**Geographic location**			0.801
Portland metro area	35,640 (78.7)	2,880 (78.5)	
Outside Portland metro area	9,633 (21.3)	787 (21.5)	

^a^Recruitable members includes 4,957 members who are represented more than once

^b^Participants include 60 members who are represented more than once

**Bold** face indicates statistical significance

### Household member participation

**Fig 4:**
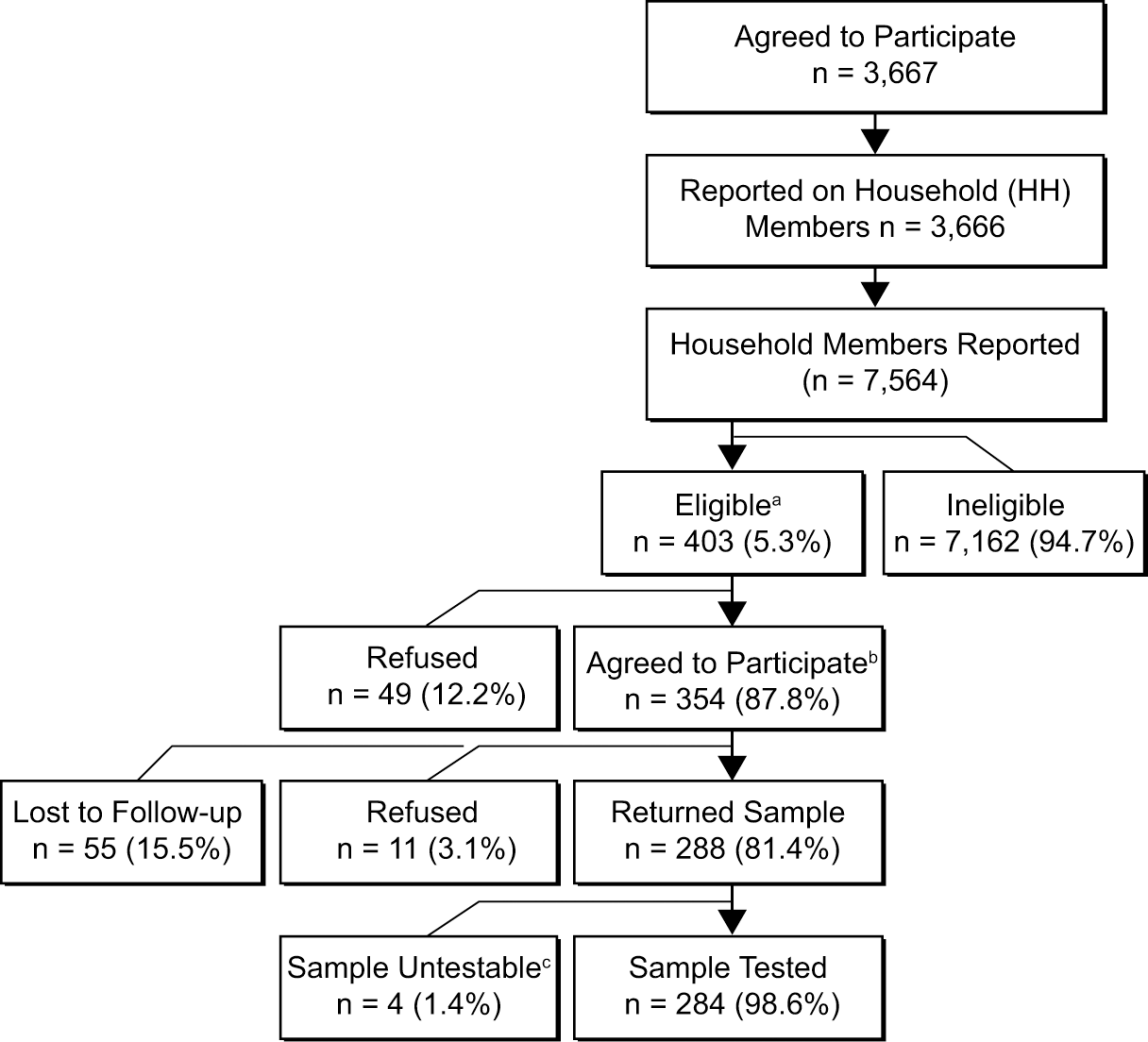
KPNW household members of enrolled MAAGE participants, April 1, 2014-September 30, 2016. ^a^Recruited household members of participants that have onset of AGE symptoms ≤7 days before primary participant’s recruitment or follow-up call. ^b^Agreed to participate and completed baseline survey. ^c^Returned samples were not viable for pathogen testing.

Information about 7,564 household members was provided by 3,666 MAAGE participants who had agreed to participate, completed the enrollment survey, and returned a stool sample (Fig 4). The majority (95%) of household members were asymptomatic within the 7-day windows prior to each survey and thus deemed ineligible for enrollment. Among 402 eligible household members, 288 (72%), representing 234 distinct households, agreed to participate and returned a stool sample.

### Specimen collection

**Fig 5:**
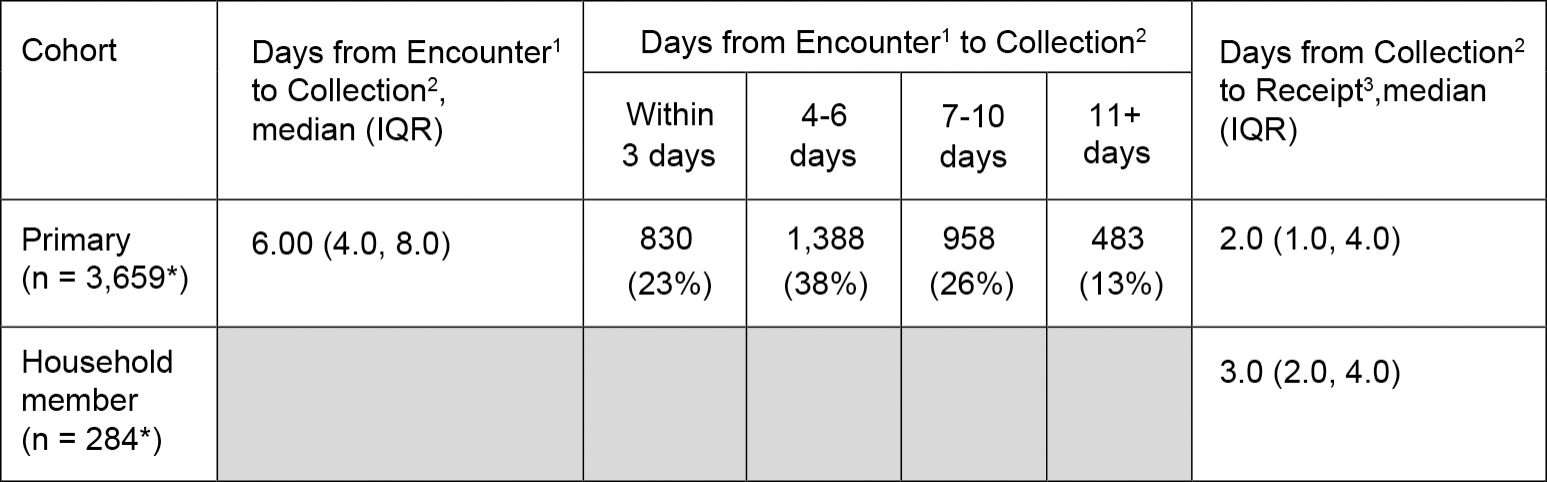
Specimen return characteristics from KPNW members enrolled in MAAGE. *Missing values (n = 8, n = 4) due to untestable stool samples. ^1^Encounter: Date of patient’s first medically-attended acute gastroenteritis (MAAGE) encounter with Kaiser Permanente Northwest (KPNW) healthcare system. ^2^Collection: Date stool sample was collected by participant. ^3^Receipt: Date sample was received by study staff at KPNW.

Of the three possible specimen return options, the majority of participants (86% of MAAGE patients and 87% of HHMs) opted to drop off their specimens at the KPNW clinical facility of their choice. Only 9% of MAAGE patients and 8% of HHMs used a courier overnight service and 5–6% of participants in each group opted to have recruitment staff pick-up the samples. Regardless of return method, we received 97% (n = 3,836) of specimens within 7 days of sample self-collection. For MAAGE patients, a median of 6.0 days (IQR: 4 days, 8 days) elapsed from the encounter to specimen collection, and an additional 2.0 days (IQR: 1 day, 4 days), from specimen collection to receipt at the CHR clinic (Fig 5*)*. For HHMs, we received specimens at the CHR clinic within a median of 3 days (IQR: 2 days, 4 days) of collection. All samples with temperature indicators were at a stable temperature upon receipt.

## Discussion

Findings from this study demonstrate the successful implementation of a novel method for conducting all-ages, population-based, active surveillance for MAAGE and its associated viral pathogens in the U.S. High rates of participation and specimen collection within an integrated health system resulted in successful recruitment of a population-based sample of 3,667 MAAGE patients and 288 symptomatic household members. These study cohorts were linked to comprehensive EHR and participant-reported survey data as well as a stool sample submitted for testing of viral AGE agents. Data collected through this study will allow for development of improved age-stratified incidence estimates of MAAGE and associated viral pathogens, and better characterization of transmission of these pathogens within households of enrolled MAAGE participants.

Use of this surveillance method could be applied to other studies seeking to develop population-based incidence rates, particularly those requiring stool sample testing from the study cohort. In anticipation of the potential introduction of norovirus vaccines, an analogous surveillance platform could be employed to monitor for vaccine effectiveness and the emergence of other AGE viruses. This methodology can be further extended to build a biobank of stool specimens and to allow for exploration in the burgeoning field of human gut microbiome research and carried antimicrobial resistance elements. Finally, this approach can also serve as a model for the active surveillance of respiratory diseases, such as influenza. This is especially relevant, as self-collection of respiratory specimens for the detection of influenza was found to be non-inferior to collection by a health care provider [15]. In all of these cases, the ability to collect representative specimens from individuals among a well-defined population linked to comprehensive EHR information is a powerful tool in the study of infectious diseases of public health importance.

This novel recruitment approach minimized potential recruitment and enrollment bias in several key ways. First, we identified cases based on any MAAGE encounter in the EHR across the entire KPNW healthcare delivery system. The fact that integrated health system members typically receive all or nearly all of their care within the system, and that even encounters that take place at contract facilities outside of the system are frequently documented and shared via data exchange, means that this approach captures nearly all cases where KPNW members sought treatment and received an AGE diagnosis, reducing the potential for selection bias based on health care utilization patterns or in-clinic recruitment efforts. Health systems that do not provide a continuum of care across all primary and specialty medical needs may not be able to access comprehensive medical encounter data to the extent that an integrated health system can. Second by conducting recruitment interviews six days per week between 9am and 7pm, we maximized our ability to recruit sampled adults who were unavailable during typical business hours. Third, recruitment calls were made from a central location using dedicated staff. The centralized call center avoided any need for staff to travel to clinics or placing undue burden of recruitment on clinical care staff. Other published studies often tie recruitment efforts to particular provider offices [16, 17] and may involve physically placing research staff within a clinic setting [16], which increases time needed for logistics planning and restricts recruitment to select clinics, which increases potential for recruitment biases. Together, these aspects of our approach maximized our ability to obtain true, population-based incidence estimates of AGE and associated viral pathogens.

Another way that our approach improved upon past methods was by recruiting participants following telephone, email, or video (i.e., remote) encounters in addition to in-person encounters. Many individuals, especially adults, do not visit a provider in person when experiencing AGE symptoms. By including this group, which accounted for 11% of our participant population, we may gain better insight into a poorly researched subset of the population experiencing AGE. These individuals are likely to have less severe disease and may be undercounted in studies of AGE incidence where recruitment occurs only during visits to healthcare providers. Further, remote encounters may occur closer to the onset of AGE symptoms compared to in-person encounters, increasing the likelihood of detecting viral pathogens in stool samples.

Unlike the present study, previous studies estimating norovirus incidence and transmission have recruited participants once they have presented for care within the health care delivery system. For example, the New Vaccine Surveillance Network (NVSN) assessed rates of MAAGE among pediatric patients and conducted specimen collection during the health encounter [7]. Similarly, the first and second Infectious Intestinal Disease study (IID and IID2) in the United Kingdom assessed rates of MAAGE among individuals of all ages following a visit to a participating general practitioner site, although stool specimens were self-collected and returned by mail [16, 17]. It seems possible that recruiting outside the delivery system would reduce participation rates, but this was not the case in our study. NVSN reported that 72% of recruited children agreed to participate, of whom 68% provided a specimen; among participants aged 5 years and younger, our study had a participation rate of 62%, with 74% of participants providing a specimen. In IID and IID2, 54% and 57%, respectively, of recruited individuals in the General Practitioner study cohorts agreed to participate and provided a specimen [7, 16–18], compared to 62% in our study (with 76% of participants returning samples). Thus, recruitment and sample collection using our methodology were comparably successful to each of these formative studies.

Although the demographic distribution of study participants was representative of the overall KPNW membership with respect to sex, encounter type, and geography, differences emerged in rates of participation by race and ethnicity. Differences by race, however, were very small (≤1%) and significance is likely due to large sample sizes. Differences by ethnicity may have been due in part to resource constraints that led to the exclusion of participants who could not speak English. Further analysis of data within these groups is needed to determine whether there are clinically significant differences between individuals experiencing AGE by race or ethnicity.

There are a few limitations to this methodology that should be noted. First, by conducting surveillance within an integrated health care delivery system, we limit participation to insured individuals who may not be representative of the general U.S. population. However, KPNW serves individuals who are on Medicare and Medicaid; 20% of KPNW members aged 0–18 years were enrolled in Medicaid in 2015 (*data unpublished*). Considering AGE data stratified by household composition and socioeconomic status measures gathered through the EHR and questionnaires can further address this issue. A second potential limitation is the exclusive use of ICD diagnostic codes for episodes of AGE. This may have resulted in missing some MAAGE cases, given the potential variability in provider assignment of diagnostic codes for events of this type. Finally, the time lag in the collection of specimens relative to symptom onset could negatively impact laboratory testing, potentially reducing our ability to accurately detect infection with specific pathogens. However, as samples were typically received within 7 days following the first AGE encounter, this should not significantly impact the study’s ability to assess AGE and associated viral pathogen incidence [19].

Initial implementation of the surveillance system for this study required extensive efforts on the part of recruitment specialists, research data analysts, and tracking system development experts, among other research team members. However, once implemented, recruitment efforts were reliant on roughly three full-time staff-equivalents able to conduct calls from a centralized location and required minimal coordination with routine clinical care staff. Future surveillance studies using these methods should be prepared to commit considerable upfront resources in order to successfully recruit, enroll, and retain participants. However, over the course of the study, we believe this strategy may be relatively cost-efficient compared to other approaches.

In conclusion, this study demonstrates the first successful implementation of an all-ages, active surveillance program for MAAGE and associated viral pathogens in the United States. The broad range of data collected in this study can lead to additional analyses focusing on incidence estimates of MAAGE and associated viral agents as well as incidence of community AGE. This novel methodology can be applied broadly to conduct rapid, active surveillance for acute infectious diseases within healthcare delivery systems that offer a comprehensive continuum of care.

## Supporting information

S1 File MAAGE index case baseline survey. (DOC)

S2 File MAAGE household member baseline survey. (DOC)

S3 File MAAGE index case two-week follow-up survey. (DOC)
