# Supplementary material for: A model for rapid, active surveillance for medically-attended acute gastroenteritis within an integrated health care delivery system
Source: PLoS One. 2018 Aug 3;13(8):e0201805. doi: 10.1371/journal.pone.0201805 (PMC6075775; doi:10.1371/journal.pone.0201805)
Supplement: S1 File — (DOC) [file pone.0201805.s001.doc]

1

**MAAGE Baseline Instrument, Index Cases**

Hello, this is _____________ from the Center for Health Research at Kaiser Permanente. May I please speak with [FIRST NAME] [LAST NAME]?

**[SECTION A—SCREENER ]**

1. We understand that you have contacted Kaiser Permanente about stomach illness. Is that correct?

Yes (continue to 2)

No (thank you for your time) [end call]

2. Have you had at least 1 episode of vomiting during this illness?

**If YES**

What was the date of your first episode?________________________

What was the date of your last episode?_______________________

OR

[if unsure] About how many days did you have vomiting? ________________

Does this include today? YES NO

**If NO** (continue to 3A)

If **Unsure/Unknown** (continue to 3A)

If **Refused/No Response** (continue to 3A)

3A. Have you had diarrhea during this illness? *Diarrhea is defined as 3 or more episodes within a 24-hour period.

**If YES**

What was the date of your first episode? ____________

What was the date of your last episode? ____________

**OR**

[if unsure] About how many days did you have diarrhea?

Does this include today? YES NO

**If NO** (continue to 3B)

If **Unsure/Unknown** (continue to 3B)

If **Refused/No Response** (continue to 3B)

* This definition for diarrhea was used throughout participant interview

3B, Do you have a medical condition that causes chronic diarrhea such as Crohn’s disease, ulcerative colitis, inflammatory bowel disease, or abdominal or colorectal cancer to name a few?

**If YES to chronic diarrhea and NO to vomiting**

[We are conducting a study with people who have acute diarrhea; due to your chronic diarrhea you are not eligible for our study. Thank you for speaking with me, have a good day]. (Screened out ever)

**If NO** to chronic diarrhea CONTINUE

**NO** [IF NO TO BOTH 2 AND 3A END THE CALL]

If **Unsure/Unknown** [IF UNSURE/UNKNOWN FOR BOTH 2 AND 3A/B END THE CALL]

If **Refused/No Response** [IF REFUSED TO BOTH 2 AND 3A/B END THE CALL]

**[ IF YES TO EITHER OR BOTH 2 OR 3A AND NOT CHRONIC CONTINUE]**

4. Are you still experiencing vomiting or diarrhea?

**YES**

**NO**

**Unsure/Unknown**

**Refused/No response**

**If yes to Q. 4 participant is asked questions in SYMPTOMATIC section in Follow Up data collection survey**

**If no to Q. 4 participant is asked questions in NON SYMPTOMATIC** section in **Follow Up data collection survey**

**[SECTION C—ILLNESS EPISODE & EXPOSURE ]**

**5. I would now like to know about your symptoms**

Did you have

Fever during this illness?

**YES**

**[if yes]** How many days ago did it begin _______Is that including today?

[**if yes**] How many days did you have a fever ______Is that including today?

Did you measure your temperature with a thermometer? [If yes] degree ____

[if no] were you warm to the touch? Y N

**NO**

Don’t know

Did you have

Headache during this illness? Y N ?

Muscle aches during this illness? Y N ?

Stomach, belly cramps during this illness? Y N ?

Unusual tired feelings during this illness? Y N ?

Shaking chills during this illness? Y N ?

Nausea during this illness? Y N ?

Any blood in stool during this illness? Y N ?

6. Did you miss work or school due to this illness?

YES [If yes] how many days,_____ is that including today Y N

NO

Don’t know n

Refused

Not Applicable

7. Did you take any medications for this illness?

YES

[If YES List]

_______________________________

_______________________________

______________________________

n 1 0 1

NO n 1

Don’t know n 1

Refused n 1

8. Did you use enhanced water or other beverages, such as pedialyte, or sport drinks with electrolytes during this illness?

If YES List

________________________________

NO

Don’t know

Refused

Ok, I am now going to ask you about the 7 days before your first symptoms showed up.

9. Did you have contact with any animal? [7 days before symptoms]

If yes, List_____________

NO

Don’t know

Refused

10. Did you travel outside the United States? [7 days before symptoms]

YES, [if yes] where?

NO

Don’t know

Refused

11. Did you eat food at restaurants, fast food, or vendors? [7 days before symptoms]

YES

NO

Don’t know n 1

Refused

12. Did you eat food prepared by others at gatherings like potlucks or events? [7 days before symptoms]

YES

NO

Don’t know n 1

Refused

13. Did you have contact with diapered children or diapered adults? [7 days before symptoms]

YES

NO

Don’t know n 1

Refused

14. Did you have contact with children in daycare or nursery school? [7 days before symptoms]

YES

NO

Don’t know n 1

Refused

15. Did you have contact with persons living in a nursing home? [7 days before symptoms]

YES

NO

Don’t know n 1

Refused

16. Did you have contact with persons with diarrhea or vomiting? [7 days before symptoms]

YES

[if yes] were these persons living outside your residence? Y N

[if yes] were these persons living inside your residence? Y N

NO

Don’t know n 1

Refused

17. Not including you, how many people live at your residence?

Amount_____[IF AMOUNT IS 0 SKIP TO Q.18]

I would like to now ask you some questions about your household members:

HOUSEHOLD MEMBER WORKSHEET

| A  age | B  sex | C  Did person have vomiting or diarrhea | D  Date symptoms began | E  Date symptoms ended | F  [If yes] was medical care sought at Kaiser? | G  Rec  Y  N | H  Persons Name F/L  (include legal  guardian  if person is  17 or younger) |
| --- | --- | --- | --- | --- | --- | --- | --- |
| 1] |  |  |  |  |  |  |  |
|  |  |  |  |  |  |  |  |
| 2] |  |  |  |  |  |  |  |
|  |  |  |  |  |  |  |  |
| 3] |  |  |  |  |  |  |  |
|  |  |  |  |  |  |  |  |
| 4] |  |  |  |  |  |  |  |
|  |  |  |  |  |  |  |  |

Based on your responses you may have household members who are eligible. May we have their name and contact information?

Yes [if yes fill out column G and H]

No [if no skip to Q.18]

18. Which category best describes your race? **[check all that apply]**

American Indian or Alaskan native __

Asian __

Black or African American __

Native Hawaiian or other Pacific Islander __

White __

Unknown/not Specified __

Refused

19. Do you identify as:

Hispanic__

Non-Hispanic__

Unknown/not Specified __

Refused

20. What is your highest level of education completed?

Less than high school __

High school or equivalent __

Some college __

College graduate __

Don’t know

Refused

21. What was your household income last year (before taxes)

Less than or equal $ 25,000

Between $25 and $75 thousand

Between $75 and $100 thousand

Between $100 and $125 thousand

Between $125 and $150 thousand

Over $150 thousand

Unknown

Refused
