# Supplementary material for: A model for rapid, active surveillance for medically-attended acute gastroenteritis within an integrated health care delivery system
Source: PLoS One. 2018 Aug 3;13(8):e0201805. doi: 10.1371/journal.pone.0201805 (PMC6075775; doi:10.1371/journal.pone.0201805)
Supplement: S2 File — (DOC) [file pone.0201805.s002.doc]

2

***Maage **Household Member Baseline Survey Instrument**

Hello, this is _____________ , I’m calling from the Center for Health Research at Kaiser Permanente. May I please speak with [FIRST NAME] [LAST NAME]?

**[SECTION A—SCREENER]**

1. We recently spoke with a household member who said you have experienced symptoms of stomach illness. Is this correct?

Yes

NO—Thank you for your time [END CALL]

2. Have you had at least 1 episode of vomiting during this illness?

**If YES**

What was the date of your first episode?________________________

What was the date of your last episode?_______________________

OR

[if unsure] About how many days did you have vomiting? ________________

Does this include today? YES NO

**If NO** (continue to 3A)

If **Unsure/Unknown** (continue to 3A)

If **Refused/No Response** (continue to 3A)

3. Have you had diarrhea during this illness? ***Diarrhea is defined as 3 or more episodes within a 24-hour period.

**If YES**

What was the date of your first episode? _______________

What was the date of your last episode?________________

**OR**

[if unsure] About how many days did you have diarrhea?

Does this include today? YES NO

**If NO** (continue to 3B)

If **Unsure/Unknown** (continue to 3B)

If **Refused/No Response** (continue to 3B)

3B. Do you have a medical condition that causes chronic diarrhea such as Crohn’s disease, ulcerative colitis, inflammatory bowel disease, or abdominal or colorectal cancer to name a few?

*Only questions for data collection and skip pattern directions were included for publication.

** This Household Member is only surveyed at baseline.

*** This definition for diarrhea was used throughout participant interview.

**If YES to chronic diarrhea and NO to vomiting**

[We are conducting a study with people who have acute diarrhea; due to your chronic diarrhea you are not eligible for our study. Thank you for speaking with me, have a good day]. (Screened out ever)

**If NO** to chronic diarrhea CONTINUE

NO **[IF NO TO BOTH 2 AND 3A END THE CALL]**

If **Unsure/Unknown** [IF UNSURE/UNKNOWN FOR BOTH 2 AND 3A/B END THE CALL]

If **Refused/No Response** **[IF REFUSED TO BOTH 2 AND 3A/B END THE CALL]**

**[ IF YES TO EITHER OR BOTH 2 OR 3A AND NOT CHRONIC CONTINUE]**

If NO thank them for their time end call.

**[SECTION C—ILLNESS EPISODE & EXPOSURE]**

4**. I would like to know about your symptoms**

Did you have

Fever during this illness?

**YES**

**[if yes]** How many days ago did it begin_______Is that including today?

[**if yes**] How many days did you have a fever,______Is that including today?

Did you measure your temperature with a thermometer? [If yes] degree ____

[if no] were you warm to the touch? Y N

**NO**

Don’t know

Did you have

Headache during this illness? Y N ?

Muscle aches during this illness? Y N ?

Stomach, belly cramps during this illness? Y N ?

Unusual tired feelings during this illness? Y N ?

Shaking chills during this illness? Y N ?

Nausea during this illness? Y N ?

Any blood in stool during this illness? Y N ?

5. Did you miss work or school due to this illness?

YES [if yes] how many days____________is the including today? Y N

NO

Don’t know n

Refused

Not Applicable

6. Did you take any medications for this illness?

YES

[If YES List]

_______________________________

_______________________________

______________________________

NO

Don’t know

Refused

7. Did you use enhanced water or other beverages, such as pedialyte, or sport drinks with electrolytes during this illness?

If YES List

________________________________

NO

Don’t know

Refused

Ok, I am now going to ask you about the 7 days before your first symptoms showed up.

8. Did you have contact with any animal? [7 days before symptoms]

If yes, List_____________

NO

Don’t know

Refused

9. Did you travel outside the United States? [7 days before symptoms]

YES, [if yes] where?

NO

Don’t know

Refused

10. Did you eat food at restaurants, fast food, or vendors? [7 days before symptoms]

YES

NO

Don’t know

Refused

11. Did you eat food prepared by others at gatherings like potlucks or events? [7 days before symptoms]

YES

NO

Don’t know

Refused

12. Did you have contact with diapered children or diapered adults? [7 days before symptoms]

YES

NO

Don’t know

Refused

13. Did you have contact with children in daycare or nursery school? [7 days before symptoms]

YES

NO

Don’t know

Refused

14. Did you have contact with persons living in a nursing home? [7 days before symptoms]

YES

NO

Don’t know

Refused

15. Did you have contact with persons experiencing vomiting or diarrhea living outside of your residence? [7 days before symptoms]

YES

NO

Don’t know

Refused

16. Which category best describes your race? **[check all that apply]**

American Indian or Alaskan native __

Asian __

Black or African American __

Native Hawaiian or other Pacific Islander __

White __

Unknown/not Specified __

Refused

17. Do you identify as:

Hispanic__

Non-Hispanic__

Unknown/not Specified __

Refused

18. What is your highest education level completed?

Less than high school __

High school or equivalent __

Some college __

College graduate __
