# Supplementary material for: A model for rapid, active surveillance for medically-attended acute gastroenteritis within an integrated health care delivery system
Source: PLoS One. 2018 Aug 3;13(8):e0201805. doi: 10.1371/journal.pone.0201805 (PMC6075775; doi:10.1371/journal.pone.0201805)
Supplement: S3 File — (DOC) [file pone.0201805.s003.doc]

3

**MAAGE Two-Week follow up instrument, Index Cases**

Hello, this is _____________ from the Center for Health Research at Kaiser Permanente. May I please speak with [FIRST NAME] [LAST NAME]?

**[SECTION A—SCREENER]**

[**IF PARTICIPANT WAS SYMPTOMATIC AT RECRUITMENT]**

1. When we spoke to you 2 weeks ago you said you were still experiencing symptoms of stomach illness.

1A. Are you still experiencing Vomiting?

**IF Yes**: skip to 1B

**IF NO**:

What was the date of your last episode of vomiting?___________

**OR**

[If unsure or don’t know for date of last vomit episode] How many days did you have vomiting since we last spoke? ________

**I did not have vomiting**

1B. Are you still experiencing diarrhea?

**If Yes**: go to Q2.

**If NO**

What was the date of your last episode of diarrhea?____________

**OR**

[If unsure or don’t know for the date of last diarrhea] how many days did you have diarrhea since we last spoke?

**I did not have diarrhea**

IF YES TO EITHER OR BOTH 1A. / 1B. GO TO Q. 2

IF NO TO BOTH 1A. AND 1B. GO TO SECTION B HOUSEHOLD MEMBERS

IF UNSURE/UNKNOWN OR REFUSED TO BOTH 1A. AND 1B. GO TO SECTION B HOUSEHOLD MEMBERS

**IF PARTICIPANT WAS NON-SYMPTOMATIC AT RECRUITMENT**

When we spoke to you 2 weeks ago you said you were no longer experiencing symptoms of stomach illness.

**1A. Have you had vomiting since we last spoke?**

**If Yes**

Since we last spoke, what was the date of your first episode of vomiting?_________

Since we last spoke, what was the date of your last episode of vomiting? __________

**OR**

[If unsure or don’t know dates of episode] Since we last spoke, how many days did you have vomiting?_____________

Does this include today? Yes NO

**If No** skip to 1B

**If unsure/unknown** skip to 1B

**If refused/No Response** skip to 1B

**1B. Have you had diarrhea since we last spoke?**

**Yes**

Since we last spoke, what was the date of your first episode of diarrhea?___________

Since we last spoke, What was the date of your last episode of diarrhea? ___________

OR

[If unsure or don’t know dates of episode] Since we last spoke how many days did you have diarrhea?_____________

__________is that including today? Yes NO

**NO**

**Unsure/Unknown**

**Refused/No Response**

IF YES TO EITHER OR BOTH 1A. / 1B. GO TO Q. 2

IF NO TO BOTH 1A. AND 1B. GO TO SECTION B HOUSEHOLD MEMBERS

IF UNSURE/UNKNOWN OR REFUSED TO BOTH 1A. AND 1B. GO TO SECTION B HOUSEHOLD MEMBERS

2. **I would like to know about your symptoms** since we last spoke.

Did you have

Fever since we last spoke?

**YES**

**[if yes]** How many days did you have a fever since we last spoke?_______

Is that including today? YES NO

[**if NO**] what was the date your fever went away?___________

Did you measure your temperature with a thermometer? [If yes] degree ____

[if no] were you warm to the touch? Y N

**NO**

Don’t know

Did you have

Headache since we last spoke? Y N ?

Muscle aches since we last spoke? Y N ?

Stomach, belly cramps since we last spoke? Y N ?

Unusual tired feelings since we last spoke? Y N ?

Shaking chills since we last spoke? Y N ?

Nausea since we last spoke? Y N ?

Any blood in stool since we last spoke? Y N ?

3. Did you miss work or school since we last spoke?

[IF YES] how many total days____________ Is this including today?

NO

Don’t know

Refused

Not applicable

4. Did you take any medications since we last spoke?

If YES List

_______________________________

No

Don’t know

Refused

5. Did you use enhanced water or other beverages, such as pedialyte, or sport drinks with electrolytes since we last spoke?

If YES List

________________________________

No

Don’t know

Refused

**SECTION B –*IF THEY HAD HOUSEHOLD MEMBERS**

6. Since we last spoke have any household members had symptoms of vomiting or diarrhea?

**Yes**

[If yes add to worksheet]

[if yes] these members may be eligible for the Household part of this study. May we have their contact number? [If yes add to WS] [if skip to end]

**NO**

[if no skip to end]

HOUSEHOLD MEMBER WORKSHEET

| A  age | B  sex | C  Did person have vomiting or diarrhea | D  Date symptoms began | E  Date symptoms ended | F  [If yes] was medical care sought at Kaiser? | G  Rec  Y  N | G  If symptoms began in the past 7 days ask for name and contact info | H  Persons Name F/L  (include legal guardian  if person is 17  or younger) |
| --- | --- | --- | --- | --- | --- | --- | --- | --- |
| 1] |  |  |  |  |  |  |  |  |
|  |  |  |  |  |  |  |  |  |
| 2] |  |  |  |  |  |  |  |  |
|  |  |  |  |  |  |  |  |  |
| 3] |  |  |  |  |  |  |  |  |
|  |  |  |  |  |  |  |  |  |
| 4] |  |  |  |  |  |  |  |  |
|  |  |  |  |  |  |  |  |  |

*Household information from the baseline instrument was electronically filled in the two week follow-up instrument. New household members could have been added at the two week follow-up.
